# Novel identified associations of *RGS1* and *RASGRP1* variants in IgA Nephropathy

**DOI:** 10.1038/srep35781

**Published:** 2016-11-02

**Authors:** Xu-Jie Zhou, Swapan K Nath, Yuan-Yuan Qi, Celi Sun, Ping Hou, Yue-Miao Zhang, Ji-Cheng Lv, Su-Fang Shi, Li-Jun Liu, Ruoyan Chen, Wanling Yang, Kevin (Zhi) He, Yanming Li, Hong Zhang

**Affiliations:** 1 Renal Division, Peking University First Hospital; Peking University Institute of Nephrology; Key Laboratory of Renal Disease, Ministry of Health of China; and Key Laboratory of Chronic Kidney Disease Prevention and Treatment (Peking University), Ministry of Education; Beijing, 100034, People’s Republic of China; 2 Arthritis and Clinical Immunology Research Program, Oklahoma Medical Research Foundation, Oklahoma City, Oklahoma, USA; 3 Department of Paediatrics and Adolescent Medicine Centre for Genomic Sciences, Queen Mary Hospital, LKS Faculty of Medicine, The University of Hong Kong, Hong Kong; 4 Department of Biostatistics, School of Public Health, University of Michigan, Ann Arbor, USA

## Abstract

Known susceptibility loci together can only explain about 6–8% of the disease heritability of IgA nephropathy (IgAN), suggesting that there are still a large number of genetic variants remained to be discovered. We previously identified IgAN and systemic lupus erythematosus (SLE)/lupus nephritis (LN) shared many loci based on GWAS on Chinese populations. The more recent study with high-density genotyping of immune-related loci in individuals with Asian ancestry identified 10 new and 6 suggestive loci in SLE. In the current study, we thus included all the lead SNPs from these 16 loci reported, and firstly tested their associations in 1,248 patients with sporadic IgAN, 737 patients with LN and 1,187 controls. Significant associations identified in IgAN were replicated in additional 500 patients and 2372 controls. rs12022418 in *RGS1 (p* = 3.0 × 10^−6^) and rs7170151 in *RASGRP1 (p* = 1.9 × 10^−5^) showed novel associations in IgAN. Compared to SNPs that were in LD with them, the associated variants showed higher potential of regulatory features by affecting gene expression. And systemic evaluation of GWAS data supported the pleiotropic effects of *RGS1* and *RASGRP1* variants in mediating human complex diseases. In conclusion, novel risk loci shared between IgAN and SLE/LN were identified, which may shed new light to exploit the potential pathogenesis for those two diseases.

IgA nephropathy (IgAN), the most common primary glomerulonephritis in the world, is a progressive disease that causes renal failure in 20–40% of patients in about 20 years^1^. Previous studies have showed strong genetic components in the pathogenesis of IgAN. Epidemiology studies suggested that the prevalence of IgAN has significant differences across ethnicity and regions, and East Asians has more IgAN than Europeans, and even more so than Africans. Familial clustering of the disease has also been well documented. To date, Genome-wide association studies (GWAS) have identified 15 loci robustly associated with IgAN with genome-wide significance (P < 5 × 10^−8^). However, all the known susceptibility loci together so far only explain about 6–8% of the disease heritability, suggesting that there are still a large number of genetic variants remained to be discovered^2^.

More recently, genetic studies have revealed highly shared immunological mechanisms in immune-related disorders, explaining the genetic basis of the shared pathogenesis of immune-related diseases^3^. Among many immune related renal diseases, IgA nephropathy (IgAN) and lupus nephritis (LN), are the two most common glomerular diseases, and are more common in East Asians than in other populations. Genetic studies have pointed out partial sharing of mechanisms of these two diseases. In our previous studies, we identified *CFH, HLA-DRA, HLA-DRB1, PXK, BLK, UBE2L3* and *MTMR3* as shared loci between IgAN and systemic lupus erythematosus (SLE)/LN based on GWAS on Chinese populations, highlighting pathways including MHC class-II antigen presentation, complement regulation, signaling by the BCR, autophagy, and ubiquitin/proteasome-dependent degradation in the pathogenesis of these two complex diseases^4^,^5^,^6^,^7^,^8^,^9^. The degree of shared genes between IgAN and SLE is substantial, but is likely to be still an underestimate because we just included candidate variants with genome-wide significance in respective GWASs and most of involved GWASs were conducted in peoples with Caucasian ancestry.

The more recent high-density genotyping of immune-related loci study in individuals with Asian ancestry identified 10 new SLE risk loci with *p* of 1.34 × 10^−9^ to 3.75 × 10^−117^ as well as 6 suggestive loci with p (1.90 × 10^−9^ to 1.12 × 10^−5^)^10^. In this international collaborative study, we analyzed more than 17,000 human DNA samples collected from four countries: China, South Korea, Malaysia and Japan. With the new data available, a further follow-up shared genetic study between IgAN and SLE is urgent deserved, especially in peoples with the same ancestry. Thus, in the current study, we conducted a follow-up shared genetic study between SLE and IgAN, including all the lead SNPs from above 16 novel loci identified from the more recent high-density genotyping of immune-related loci study in individuals with Asian ancestry, and tested their associations in more than 6,000 northern Chinese individuals.

## Results

### Associations between novel lead SNPs newly identified in Asian SLE and susceptibility to IgA nephropathy

In the current study, as introduced in the method, 21 SNPs representing 16 SLE risk loci were firstly genotyped in 1,248 patients with sporadic IgAN and 1187 unrelated healthy controls. After quality control, 20 SNPs for 1148 cases and 1166 controls were left for association analysis. Among the 20 variants from 15 loci analyzed, 2 variants were associated with susceptibility to IgA nephropathy, including rs12022418C in *RGS1* (9.7 kb 5′ of *RGS1*) with *p* of 6.27 × 10^−4^ (OR 1.24, 95% CI 1.10–1.40) and rs7170151C in *RASGRP1* (intronic) with *p* of 8.34 × 10^−4^ (OR 1.22, 95% CI 1.09–1.37) (Table 1). Although the significance was still far from genome-wide significance of *p* < 5 × 10^−8^, they can survive in multiple testing in candidate variant associations (*p* threshold of 2.5 × 10^−3^ for 20 candidate variants), suggesting highly likely associations. And in the current study, assuming the odds ratio of 1.25, we had the power of 0.95 and 0.96 for rs12022418 and rs7170151 respectively. For other variants, rs2009453T, rs931127G in *PCNXL3* and rs11235604T in *ATG16L2-FCHSD2* loci showed nominal association signal with *p* approaching 0.05. In genetic model fitting, the recessive model of rs12022418 showed a 67% increased risk among patients carrying CC genotypes compared to controls (14.5% vs. 9.3%, OR 1.67, 95% CI 1.28–1.40, *p* = 1.24 × 10^−4^). Whereas the allelic models of rs7170151, rs931127and rs11235604 remained the best fit in their respective fittings (genotypic, trend, allelic, dominant, and recessive models).

### Most of the novel SLE associated SNPs indentified could be replicated in our LN cohort

As a replication in SLE, no multiple testing was applied and a *p* value < 0.05 was considered significant. As could be referred in Table 1, seven out of 20 SNPs in *TCF7, GTF2I- GTF2IRD1, RASGRP1, CD226 and SYNGR1* have been replicated with *p* values ranging from4.27 × 10^−2^ to 5.58 × 10^−29^, while three other variants in *TERT, LOC285627-IL12B, and SIGLEC6* showed nominal associations (with p of 0.05~0.08). All the associated variants showed the similar risk effects as previously reported. Of special note, although the current sample size may be still underpowered, variants locating *GTF2I-GTF2IRD1*showed the genome-wide associations with p < 5 × 10^−8^ and with high altitude of effect size (OR > 2), highlighting important etiology role of *GTF2I-GTF2IRD1* in SLE.

### Associations by regional analysis with previous GWAS data

**Figure 1:**
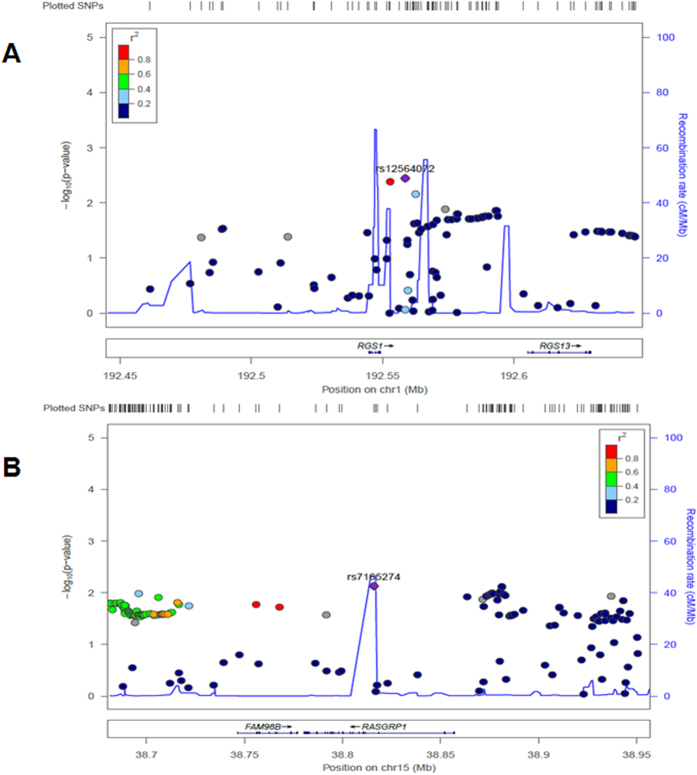


To validate the associations for the two likely risk loci in IgA nephropathy, we retrieved the regional genotype data including 100 kb upstream and downstream of the gene. In the original GWAS data, *RGS1* rs10489871 (2.5 kb 3′ of RGS1, p = 4.83 × 10^−2^) and *RASGRP1* rs4923802 (24 kb 5′ of *RASGRP1*, p = 7.91 × 10^−3^) showed the most significance. With imputation data after quality control, as can be seen from Fig. 1, *RGS1* rs12564072 (9.5 kb 3′ of *RGS1, p* = 3.60 × 10^−3^), *RGS1* rs77066951 (3.8 kb 3′ of *RGS1*,*p* = 4.20 × 10^−3^) showed significant associations with IgA nephropathy (exceeding P < 5 × 10^−3^), and *RASGRP1*rs7165274 (intronic, *p* = 7.52 × 10^−3^) showed nominal association signal with *p* approaching 5 × 10^−3^. Among the associated variants, rs77066951 was in high LD with rs12564072 (r^2^ = 0.94). However, due to low imputation quality, we failed to impute the two lead variants, namely rs12022418 in *RGS1* and rs7170151 in *RASGRP1* (Rsq < 0.1).

### Independent genetic replications of RGS1 and RASGRP1 variants

Direct genetic replications for *RGS1* rs10489871 and *RASGRP1* rs4923802 were conducted in further in an additional cohort comprised of 500 patients with IgAN and 2372 controls. As can be seen from Table 2, the associations between *RGS1* rs12022418 (*p* = 2.88 × 10^−3^), *RGS1* rs7170151 (*p* = 9.05 × 10^−3^) and susceptibility to IgA nephropathy could be independently replicated. And the associations were enhanced with combined data in a total of 1,748 patients with sporadic IgAN, and 3,559 controls. The OR values for rs12022418C in *RGS1 (p* = 3.0 × 10^−6^) and rs7170151C in *RASGRP1 (p* = 1.9 × 10^−5^) were 1.23 and 1.20 respectively.

### Functional annotation of RGS1 and RASGRP1 variants

It’s reported that the lead SNPs associated are not necessarily the causal ones, since there are often SNPs strongly correlated to GWS SNPs due to high LD. Therefore, we extracted proxy SNPs (r^2^ ≥ 0.8) to rs12022418 and rs7170151 for functional annotation using RegulomeDB. For rs12022418, locating in 9.7 kb 5′ of *RGS1*, no other proxy SNP was identified. It was annotated as regulatory with “score 5: minimal binding evidence”. Using the HaploReg v4.1, we observed that rs12022418 was indeed residing in a region overlapping promoter histone marks in stem cells (ESDR) and enhancer histone marks in blood cells (BLD). And rs12022418 was observed to be an eSNP (expression regulatory SNP) associating with altered expression levels of *RGS1* (eQTL) in the Genotype-Tissue Expression (GTEx) pilot analysis. For rs7170151, 7 other proxy SNPs were identified (rs8032939, rs8035957, rs4924273, rs6495979, rs11348849, rs11631591, rs7173565) and all were intronic ones. Among the eight proxy SNPs, rs7170151 and rs11631591 had the highest evidence for function (score 3a: TF binding + any motif + DNase peak). In HaploReg v4.1, all the 8 SNPs were annotated eSNPs, indicating that *RASGRP1* variants are likely to influence disease through mechanisms regulating gene expression. In addition, it had been reported that rs8032939 associated with rheumatoid arthritis and rs8035957 associated with type 1 diabetes in several GWASs (the NHGRI-EBI GWAS Catalog, http://www.ebi.ac.uk/gwas), suggesting *RASGRP1* variants were pleiotropic and likely causal variants affecting multiple autoimmune diseases susceptibility.

### Pleiotropic Effect of RGS1 and RASGRP1 variants

By a systematic evaluation of the pleiotropic effects of *RGS1* and *RASGRP1* variants by searching for reported associations of these loci with other diseases in the GWAS databases, we observed their high relevance to human complex diseases. As referred in Supplementary Table 1, both 40 variants were suggested to be *RGS1* and *RASGRP1* variants impacting susceptibility to immune related diseases, metabolic diseases, psychiatric diseases, and cancer. The most majority of these variants were locating in flanking regions, with only 1 variant of *RGS1* and 7 variants of *RASGRP1* were intronic ones, suggesting highly regulatory potentials of the associated variants. Of special note, rs6428106 (5.3 kb 3′ of *RGS1*) was suggested to be associated with chronic kidney disease among participants of African ancestry. Both *RGS1* and *RASGRP1* variants associated with systemic lupus erythematosus, Crohn’s disease, rheumatoid arthritis, multiple sclerosis, blood pressure, type 1 diabetes, type 2 diabetes, urinary metabolites and response to chemotherapy.

**Figure 2:**
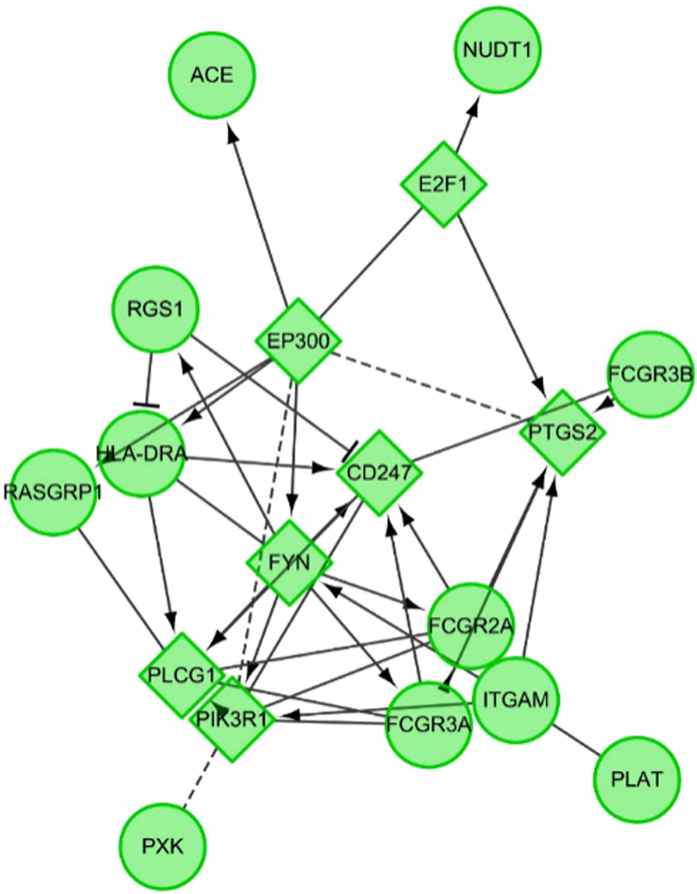


When integrating identified novel molecules in a Cytoscape-supported network involvement, it indicated that the identified two novel genes associated with these reported “old” genes (Supplementary Table 1), suggesting their functional relevance in disease pathogenesis (Fig. 2).

## Discussion

In the current study, with a salutary lesson from shared genetics among multiple immune-related diseases, we took a follow-up shared genetic study between SLE and IgAN in Northern Han Chinese. We selected lead SNPs identified in more recent large-scale high-density genotyping of immune-related loci study in individuals with Asian ancestry. We observed novel associations of *RGS1* and *RASGRP1* variants in IgA Nephropathy.

*RGS1*^11^, expressed in immune cells including B lymphocytes, T lymphocytes, natural killer (NK) cells, dendritic cells (DCs), and monocytes, belongs to the regulator of G protein signaling (RGS) family, and multiple evidence indicate that G protein deficiency or RGS resistance leads to immune abnormalities. Previous studies showed that *RGS1* regulates B cell homing to lymph nodes and motility within the lymph nodes microenvironment by regulating chemokine signaling. *RGS1*-deficient B cells exhibited increased chemotaxis and accentuated and prolonged Ca2+ mobilization responses to chemokines such as CXCL12. In Gnai2^−/−^ mice developing spontaneous inflammatory bowel disease, gut-derived T cells from Rgs1^−/−^ mice displayed increased chemotaxis to CXCL12, suggesting that the balance between expression of Gαi2 and *RGS1* in gut T cells may control their retention within the gastrointestinal mucosa. And *RGS1*-deficient macrophages showed poor retention to atherosclerotic plaques. In addition, a growing number of large GWAS in humans have identified a link between polymorphic variants in Rgs1 and chronic inflammatory diseases including celiac disease, multiple sclerosis, and type I diabetes. Although definitive mechanistic studies of *RGS1* in these diseases have yet to be performed^12^, some findings suggest that high expression of *RGS1* could promote retention of proinflammatory cells. In our current study, we observed rs12022418 in *RGS1* (9.7 kb 5′ of *RGS1*) associated with IgAN, and rs12022418 associated with altered expression levels of *RGS1* (eQTL) in the Genotype-Tissue Expression (GTEx) pilot analysis, possibly due to its location in lymphocyte regulatory regions.

*RASGRP1* is a member of a family of genes characterized by the presence of a Ras superfamily guanine nucleotide exchange factor (GEF) domain. It functions as a diacylglycerol (DAG)-regulated nucleotide exchange factor specifically activating Ras through the exchange of bound GDP for GTP. It activates the Erk/MAP kinase cascade and regulates T-cells and B-cells development, homeostasis and differentiation. Previous studies suggested that chronic T cell immunodeficiency in RasGRP1^(−/−)^ mice may be responsible for CD4 T cell activation, proliferation, and exhaustion^13^, and autoreactive B cells lacking Rasgrp1 break central and peripheral tolerance^14^. Mice deficient in Rasgrp1 develop a lymphoproliferative disorder with features of human systemic lupus erythematosus and altered expression of the different isoforms of Rasgrp1 may be a cause of susceptibility to systemic lupus erythematosus (SLE)^15^. More recent genetic study had confirmed such associations. In the current study, we observed novel associations between *RASGRP1* variants in IgA Nephropathy. rs7170151 had the highest evidence for function among SNPs that were in LD with it, all the SNPs in LD were annotated eSNPs, further suggesting defective expression of *RASGRP1* may be key factor in mediating autoimmunity.

In line with the common role of *RGS1* and *RASGRP1* in lymphocyte homeostasis, we carried out a systematic evaluation of the pleiotropic effects by searching for reported associations of these loci with other diseases. And we indeed observed their pleiotropic effect by affecting susceptibility to multiple complex diseases, and many of them showed genome-wide significance and can be replicated by other independent studies, indicating their association were not by chance. Of special note, one *RGS1* variant rs6428106 was reported to be associated with chronic kidney disease(CKD) among participants of African ancestry^16^. Although IgAN is comparatively rare in Africans, it would be of further interest to determine its correlations with the exact cause of CKD.

The genetic study of immune-related diseases has only revealed the tip of the iceberg thus far, as more genes need to be found and the causal variants need to be identified. Nevertheless, the notion of shared genetic pathways creates new and powerful approaches for discovering the full repertoire of susceptibility genes because genetic resources can be shared instead of focusing on single diseases. For example, performing meta-analyses across multiple immune-related diseases might be a powerful method for discovering more of the common genetic variation that contributes to the pathways that are shared in immune disorders, which is underway.

We first focused on the two most common causes of glomerular diseases, IgA nephropathy and lupus nephritis, which are more common in East Asians than in other populations. Genetic studies have pointed out partial sharing of mechanisms of these two diseases. Of special note, most of their shared susceptibility allele effect in opposite directions– increases risk for SLE but is protective for IgA nephropathy (‘correlated but discordant’).This study promises identification of novel susceptibility genes for IgAN by follow-up shared genetics with SLE. The observation that the associated allele of RGS1 was the reverse to that reported previously for SLE was in accordance with reports stating that the majority of protective alleles for IgAN had been implicated as risk factors for SLE. Although, genetic studies that will enhance our understanding of the links between IgAN and SLE are still on the horizon, investigating genetic variants associated with such strategy may help us understand the shared and different mechanisms involving the two common glomerular diseases s and modify future targeted therapy.

In conclusion, we observed novel associations of *RGS1* and *RASGRP1* variants in IgA Nephropathy. Compared to SNPs that were in LD with them, the associated variants showed higher potential of regulatory features by affecting gene expression. And systemic evaluation of GWAS data supported the pleiotropic effects of *RGS1* and *RASGRP1* variants in mediating human complex diseases.

### Samples and Methods

#### SNP selection and genotyping

A total of 16 lead SNPs were selected for the association study^10^, but only the assays for 13 were successfully designed for multiplex genotyping analysis by Sequenom. The three SNPs were rs73366469 in *GTF2I*, rs7726159 in *TERT* and rs12900339 in *RASGRP1*. Thus selected SNPs that were in high linkage disequilibrium with the lead SNPs using data from Asian peoples in 1000 genomes pilot 1 project with r^2^ threshold 0.8, were selected by SNAP software version 2.2 (https://www.broadinstitute.org/mpg/snap/index.php). At last, 21 SNPs representing 16 SLE risk loci were included and genotyping was performed using the MassArray system from Sequenom. For replications of selected two SNPs in additional independent cohort, TaqMan assays were conducted as previously reported^6^,^7^.

#### Study population

In the current study, we firstly recruited 3,172 northern Chinese individuals, including 1,248 patients (40.3 ± 13.1 years, 61.5% males) with sporadic IgAN (diagnosed from 2009 to 2013), 737 patients (32.7 ± 12.7 years, 83.5% females) with sporadic LN, and 1187 age and geographically (36.2 ± 13.4 years, 52% males) matched unrelated healthy controls during the same time interval. All the patients and controls were self-reported Han Chinese living in North of China for generations. The diagnosis of IgAN was proven by renal biopsy on the basis of granular deposition of IgA in the glomerular mesangium by immunofluorescence detection and the deposition of electron-dense material in the mesangium by ultrastructural examination, in which Henoch–Schonlein purpura, SLE, and chronic hepatic diseases were excluded by detailed clinical and laboratory examinations. The diagnosis of LN firstly met the revisedAmerican College of Rheumatology (ACR) classification of SLE and renal biopsies were conducted in all LN patients by light microscopy, immunofluorescence,and electron microscopy to confirm LN diagnostic criteria.

To confirm the genetic associations, regional genotypic data from GWAS conducted in 1,194 IgAN cases and 902 healthy controlsof Northern Chinese Han ancestry were also examined. Imputations were conducted using IMPUTE2 with HapMap JPT + CHB samples as the reference panel. And direct genetic replications for *RGS1* rs10489871 and *RASGRP1* rs4923802 were conducted in further in an additional cohort comprised of 500 patients with IgAN and 2372 controls.

The study protocol was approved by the Medical Ethics Committee of Peking University, and informed written consent was obtained from all patients. All procedures for genotyping were carried out in accordance with the approved guidelines.

#### Pleiotropic effect analysis of associated variants

To further highlight evidence supporting a common genetic basis of associated variants in immune disease, we carried out a systematic evaluation of the pleiotropic effects by searching for reported associations of these loci with other diseases in the databases including the NHGRI-EBI GWAS Catalog (http://www.ebi.ac.uk/gwas), Phenotype-Genotype Integrator (PheGenI) (www.ncbi.nlm.nih.gov/gap/PheGenI/) and GWASdb v2 (http://jjwanglab.org/), which provide robust lookup for published GWAS and meta-analysis studies.

In addition, to add novel findings in the context of other reported loci shared between IgAN and SLE, we searched the reported literatures using items of “IgA nephropathy AND lupus AND variant” or “IgA nephropathy AND lupus AND gene” or “IgA nephropathy AND lupus AND SNP” or “IgA nephropathy AND lupus AND polymorphism” and retrieved 16 genes likely associated with both IgAN and SLE. And to interpret the biological relevance of these genes, we obtained the interaction network of the input gene by Cytoscape with JEPETTO and Reactome plugin.

### Data quality control aND statistical analysis

For all SNPs, we examined the clustering patterns of genotypes and selected mass peaks and confirmed that the genotype calls were of good quality (rs10807150 was excluded due to low quality call). In quality control filtering, all SNPs were with call rates of >95%, HWE P > 0.001 in cases/controls, and no significantly different genotype call rates between cases and controls. Samples with call rates of <90% were removed from analysis. After quality control, 20 SNPs for 2314 individuals (1148 IgAN cases, 686 LN cases and 1166 controls) were left for further analysis. Power of the study was calculated by Power and Sample Size Calculations Software v3.1.2 (http://biostat.mc.vanderbilt.edu/PowerSampleSize). Genotype frequencies between cases and controls were compared using PLINK v1.9 (http://pngu.mgh.harvard.edu/~purcell/plink/plink2.shtml). Genetic models were defined relative to the minor allele. For multiple comparisons, both Bonferroni method and permutation procedurewith empirical distribution with resolution of 10000 were applied.

## Additional Information

**How to cite this article**: Zhou, X.-J. *et al*. Novel identified associations of *RGS1* and *RASGRP1* variants in IgA Nephropathy. *Sci. Rep.* **6**, 35781; doi: 10.1038/srep35781 (2016).

**Publisher’s note:** Springer Nature remains neutral with regard to jurisdictional claims in published maps and institutional affiliations.

## Supplementary Material

Supplementary Information

## Figures and Tables

**Table 1:** Association between new SLE risk variants inNorthern Han Chinese with IgA nephropathy or lupus nephritis.

Chr	SNP	Genes/Loci	dbSNP Annotation	BP (hg19)	Minor allele	MAF control (%)	IgAN				LN			
							MAF case (%)	P (P_permutation_)	OR	95% CI of OR	MAF case (%)	P (P_permutation_)	OR	95% CI of OR
1	rs12022418	*9.7* *kb 5*′ *of RGS1*		192535106	C	31.67	36.48	**6.27** × **10**^**−4**^**(1.27** × **10**^**−2**^)	**1.24**	1.10–1.40	32.57	0.58	1.04	0.90–1.20
1	rs7556469	*PTPRC*	intronic	198637581	G	14.5	15.33	0.43	1.07	0.91–1.26	13.28	0.31	0.90	0.74–1.10
3	rs10936599	*MYNN*	synonymous	169492100	C	45.29	45.41	0.93	1.01	0.89–1.13	47.26	0.25	1.08	0.95–1.24
3	rs10936600	*LRRC34*	missense	169514584	A	45	45.15	0.92	1.01	0.87–1.13	46.67	0.33	1.07	0.93–1.22
5	rs2736100	*TERT*	intronic	1286515	G	41	39.51	0.3	0.94	0.84–1.06	44.28	**0.05 (0.067)**	1.14	1.00–1.31
5	rs7726414	*19* *kb 5*′ *of TCF7*		133431833	T	6.36	6.38	0.99	1.00	0.79–1.27	8.53	**1.52** × **10**^**−2**^**(1.45** × **10**^**−2**^)	1.37	1.06–1.76
5	rs2421184	*LOC285627-IL12B*	intronic	158886938	A	44.26	44.22	0.98	1.00	0.89–1.12	47.19	0.08	1.13	0.98–1.29
7	rs11981999	*GTF2I*	intronic	74002082	A	34.8	32.76	0.14	0.91	0.81–1.03	43.91	**4.51** × **10**^**−8**^ **(1** × **10**^**−6**^)	1.47	1.28–1.68
7	rs117026326	*GTF2IRD1*	intronic	74126033	T	15.88	14.13	0.1	0.87	0.74–1.03	31.65	**5.58** × **10**^**−29**^**(1** × **10**^**−6**^)	2.45	2.09–2.88
11	rs2009453	*PCNXL3*	intronic	65399527	T	42.97	40.09	0.05	0.89	0.79–1.00	41.11	0.27	0.93	0.81–1.06
11	rs931127	*PCNXL3*		65405299	A	43.61	40.83	0.06	0.89	0.79–1.00	41.41	0.19	0.91	0.80–1.05
11	rs11235604	*ATG16L2*	missense	72533535	T	10.31	8.68	0.06	0.83	0.68–1.01	9.64	0.52	0.93	0.74–1.16
11	rs11235667	*FCHSD2*		72863696	G	10.47	9.03	0.1	0.85	0.70–1.03	9.41	0.30	0.89	0.71–1.11
13	rs1885889	*UBAC2-MIR548AN*		100091299	T	37.88	35.38	0.08	0.90	0.80–1.01	38.22	0.84	1.02	0.88–1.16
15	rs7170151	*RASGRP1*	intronic	38846677	C	44.47	49.39	**8.34** × **10**^**−4**^**(6.46** × **10**^**−4**^)	**1.22**	1.09–1.37	41.04	**4.27** × **10**^**−2**^**(0.05)**	**0.87**	0.76–0.99
16	rs223881	*6.1* *kb 5*′ *of CCL22*		57386565	T	49.35	48.73	0.67	0.98	0.87–1.10	51.11	0.30	1.07	0.94–1.23
18	rs1610555	*CD226*	intronic	67543146	T	30.48	31.86	0.31	1.07	0.94–1.21	33.85	**3.42** × **10**^**−2**^**(4.05** × **10**^**−2**^)	1.17	1.01–1.35
18	rs1788097	*CD226*	intronic	67543687	T	33.87	35.27	0.32	1.06	0.94–1.20	37.26	**3.78** × **10**^**−2**^**(4.30** × **10**^**−2**^)	1.16	1.01–1.33
19	rs2305772	*SIGLEC6*	missense	52033741	A	45.29	43.74	0.29	0.94	0.84–1.06	42.37	0.08	0.89	0.78–1.02
22	rs61616683	*SYNGR1*	intronic	39755772	C	21.44	22.37	0.45	1.06	0.92–1.21	18.37	**2.58** × **10**^**−2**^**(2.93** × **10**^**−2**^)	0.82	0.70–0.98

CI, confidence interval; Chr, chromosome; MAF, minor allele frequency; OR, odds ratio; IgAN, immunoglobulin-A nephropathy; SLE, systemic lupus erythematosus; SNP, single nucleotide polymorphism.

OR values were calculated basing on minor alleles for comparison. *Reported OR values for SLE were derived from the meta-analysis data reported and the lead SNPs passing quality control were listed.

Only SNPs in RGS1 and RASGRP1 loci could retain statistically significant after multiple correction, which were marked in bold.

P_permutation_ values based on 10000 permutations.

**Table 2:** Independent genetic associations of *RGS1* and *RASGRP1* variants in IgA nephropathy.

Chr	SNP	IgAN discovery				IgAN replication				IgAN in total		
		MAF case/control (%)	P	OR	95% CI of OR	MAF case/control (%)	P	OR	95% CI of OR	P	OR	95% CI of OR
1	rs12022418	36.48/31.67	**6.27** × **10**^**−4**^	**1.24**	1.10–1.40	37.00/32.13	2.88 × **10**^**−3**^	1.15	1.05–1.26	3.00 × **10**^**−6**^	1.23	1.13–1.34
15	rs7170151	49.39/44.47	**8.34** × **10**^**−4**^	**1.22**	1.09–1.37	49.80/45.27	**9.05** × **10**^**−3**^	**1.20**	1.05–1.38	1.90 × **10**^**−5**^	1.20	1.10–1.30

Discovery cohort: 1,248 patients with sporadic IgAN, and 1,187 controls.

Replication cohort: 500 patients with sporadic IgAN, and 2,372 controls.
